# A genome-wide scan for type 1 diabetes susceptibility genes in nuclear families with multiple affected siblings in Finland

**DOI:** 10.1186/1471-2156-8-84

**Published:** 2007-12-19

**Authors:** Qing Qiao, Anne-May Österholm, Bing He, Janne Pitkäniemi, Heather J Cordell, Cinzia Sarti, Leena Kinnunen, Eva Tuomilehto-Wolf, Karl Tryggvason, Jaakko Tuomilehto

**Affiliations:** 1 Department of Public Health, University of Helsinki, Finland; 2 Diabetes Unit, Department of Epidemiology and Health Promotion, National Public Health Institute, Helsinki, Finland; 3 Division of Matrix Biology, Department of Medical Biochemistry and Biophysics, Karolinska Institute, Stockholm, Sweden; 4 Institute of Human Genetics, Newcastle University, UK; 5 South Ostrobothnia Central Hospital, Seinäjoki, Finland

## Abstract

**Background:**

A genome-wide search for genes that predispose to type 1 diabetes using linkage analysis was performed using 900 microsatellite markers in 70 nuclear families with affected siblings from Finland, a population expected to be more genetically homogeneous than others, and having the highest incidence of type 1 diabetes in the world and, yet, the highest proportion in Europe of cases (10%) carrying neither of the highest risk *HLA *haplotypes that include DR3 or DR4 alleles.

**Results:**

In addition to the evidence of linkage to the *HLA *region on 6p21 (nominal p = 4.0 × 10^-6^), significant evidence of linkage in other chromosome regions was not detected with a single-locus analysis. The two-locus analysis conditional on the *HLA *gave a maximum lod score (MLS) of 3.1 (nominal p = 2 × 10^-4^) on chromosome 9p13 under an additive model; MLS of 2.1 (nominal p = 6.1 × 10^-3^) on chromosome 17p12 and MLS of 2.5 (nominal p = 2.9 × 10^-3^) on chromosome 18p11 under a general model.

**Conclusion:**

Our genome scan data confirmed the primary contribution of the *HLA *genes also in the high-risk Finnish population, and suggest that non-*HLA *genes also contribute to the familial clustering of type 1 diabetes in Finland.

## Background

Type 1 diabetes is the third most prevalent chronic disease of childhood, affecting 0.4% of the general population by age of 30 years and has a lifetime risk of nearly 1% [1,2]. The incidence of type 1 diabetes varies very significantly between populations [1,3-6]. A 30-fold difference in type 1 diabetes risk has been detected worldwide with the highest incidence of the disease in Finland and the lowest incidence of the disease in Asia [4-7]. The etiology of type 1 diabetes is unknown, but it is recognized to be due to both genetic and environmental determinants [8,9].

The observation of familial clustering of type 1 diabetes suggests that genetic factors are involved in the etiology of type 1 diabetes. For people of European ancestry, the frequency of type 1 diabetes in siblings of affected individuals is about 6% by the age of 30 [10,11], while the frequency in the general population is about 0.4–0.5% by the age of 30 [2]. Thus, type 1 diabetes is about 15 times (6/0.4) more common in siblings of type 1 diabetic patients than in the general population. The risk of type 1 diabetes increased also in the offspring of diabetic patients, to about 3–6% [12]. The concordance rate for type 1 diabetes has been found to be higher in monozygotic (MZ, 100% shared genes) than in dizygotic (DZ, average 50% shared genes) twins, with cumulative MZ rates approximately 35–50% [13-18]. The population-based Finnish [18] and Danish [17] studies have revealed that genetic factors may contribute approximately 75–80% of the liability to type 1 diabetes. The high discordance between MZ twins, however, suggests that the penetrance of the type 1 susceptibility genes is low.

Following the lead from gene identification studies in rare Mendelian diseases and the clear evidence of linkage of the MHC in human and mouse to type 1 diabetes, genome-wide scans for linkage to type 1 diabetes were undertaken [19-26]. These studies all confirmed the importance of *HLA*-encoded susceptibility to type 1 diabetes (designated also as IDDM1); and also excluded the possibility of a locus with an effect equivalent to *HLA*. The individual impact of other susceptibility genes is, therefore, much smaller than that of *HLA*. Nevertheless, statistically significant and suggestive evidence of linkage of type 1 diabetes to at least ten chromosome regions has been published, although association studies at *INS *[27] and *CTLA4 *have been required to confirm with fine map loci. These newly identified susceptibility loci showed evidence for linkage in some studies but could not be replicated in others. The discrepancies between the studies may be due to a number of factors, including sample size, genetic and phenotypic heterogeneity between data sets, genotyping methods, gender-specific effects and genetic epistasis [12,28]. We are now reporting here the first genome scan results using microsatellite markers among Finnish nuclear families with at least two siblings affected with type 1 diabetes. Finland is known to have a high degree of population homogeneity genetically, compared with most other countries because it has a distinct founder population. Finnish type 1 diabetic patients have a high frequency of non-DR3, non-DR4 positive *HLA *haplotypes (approximately 10%), suggesting the possibility that non-*HLA *loci, perhaps with a reasonable penetrance and population allelic frequency due to founder effects exists in Finland.

## Results

**Figure 1:**
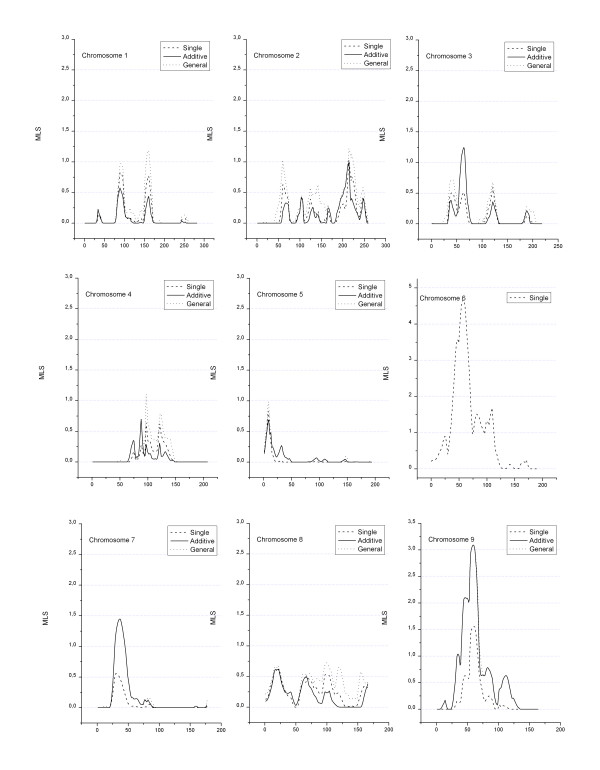
**Genome-wide linkage analysis of type 1 diabetes (Chromosomes 1–9)**. MLS resulted from the two-locus linkage analysis using TWOLOCARP in all affected sib pairs. Genetic distance (in Kosambi centi-Morgans) is given along the X-axis.

**Figure 2:**
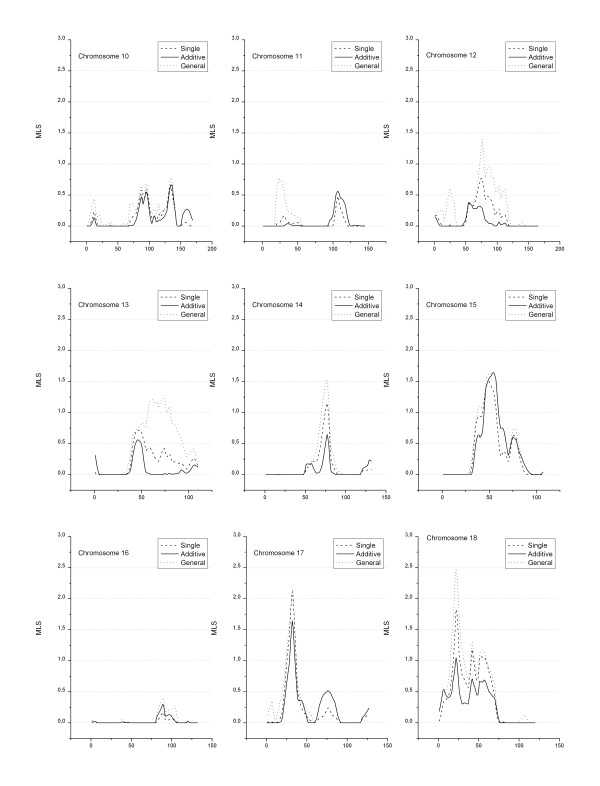
**Genome-wide linkage analysis of type 1 diabetes (Chromosomes 10–18)**. MLS resulted from the two-locus linkage analysis using TWOLOCARP in all affected sib pairs. Genetic distance (in Kosambi centi-Morgans) is given along the X-axis.

**Figure 3:**
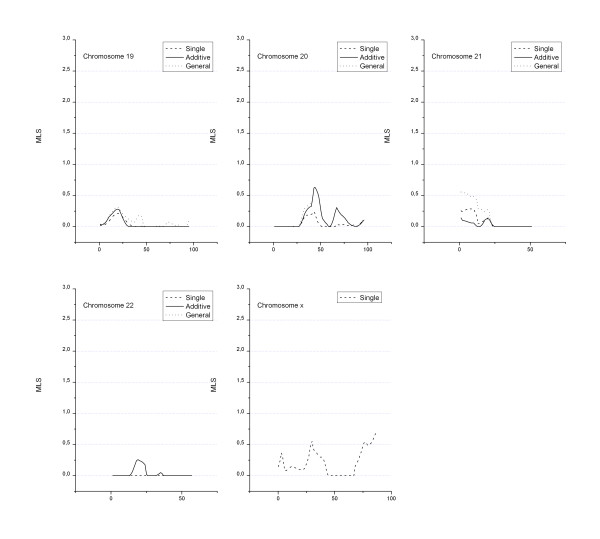
**Genome-wide linkage analysis of type 1 diabetes (Chromosomes 19–22 and Chromosome X)**. MLS resulted from the two-locus linkage analysis using TWOLOCARP in all affected sib pairs. Genetic distance (in Kosambi centi-Morgans) is given along the X-axis.

A total of 868 loci with a length of 3518 cM were actually genotyped. Figures 1, 2, 3 show the MLS curves generated using a single-locus analysis or a two-locus analysis fitted under additive and general models for the action of the two loci. The highest MLS was at chromosome 6p21, the *HLA *region where the major type 1 diabetes susceptibility gene(s) locates. For the two-locus analysis, we fixed markers with highest MLS at the *HLA *region to adjust for the effect of *HLA*. Then the joint IBD sharing at the *HLA *locus and at a second locus was considered, which was placed at an increment of 1.0 cM across the genome. The MLS for the effect of *HLA *was subtracted so that the curves in figures represent the additional contribution of locus 2. As expected, at positions unlinked to *HLA *such as those on different chromosomes, the multiplicative curves were identical to those curves obtained by use of a single-locus model for locus 2 and therefore were not presented in the figures.

For a single-locus analysis, a MLS of 4.67 (nominal p = 4.0 × 10^-6^) at chromosome 6p21 (*HLA *region) was observed. Another region, on 9p13 shows a MLS of 3.09 under either a two-locus additive model (nominal p = 2 × 10^-4^) or general model (nominal p = 4 × 10^-4^). Two other regions on 17p12 and 18p11 showed positive findings under two-locus general model (Table 1), with MLS of 2.1 and 2.5, respectively (which remains suggestive even after the Bonferroni correction for the three tests performed – single/multiplicative, additive and general – is applied).

**Table 1:** Regions with MLS over 1.0 in either single- or two-locus linkage analysis

Chromosome	Closest marker	Location (cM)	MLS (nominal p value)		
			
			Single	Two-Locus Conditional on 6p21	
				
				Additive	General
1q23	D1S1653	160	0.77 (0.0467)	0.43 (0.1240)	1.19 (0.0470)
2q34-36	D2S163	214	1.03 (0.0240)	0.99 (0.0327)	1.21 (0.0443)
3p21	D3S1581	64	0.51 (0.0935)	1.25 (0.0173)	1.25 (0.0407)
4q21	D4S1534	97	0.64 (0.0658)	0.29 (0.1876)	1.10 (0.0564)
**6p21**	**D6S1575**	**58**	**4.67 (0.000004)**	**NA NA**	**NA NA**
6q13	D6S1557	82	1.52 (0.0071)	0.06 (0.4040)	0.06 (0.5990)
6q16	D6S1709	109	1.68 (0.0048)	0.97 (0.0260)	0.97 (0.0770)
7p15	D7S516	36	0.49 (0.0987)	1.45 (0.0112)	1.45 (0.0277)
**9p13**	**D9S1817**	**59**	**1.56 (0.0064)**	**3.09 (0.0002)**	**3.09 (0.0004)**
12q14	D12S83	76	0.77 (0.0467)	0.29 (0.1876)	1.40 (0.0294)
13q32	D13S1298	74	0.43 (0.1167)	0.02 (0.4667)	1.23 (0.0428)
14q24	D14S986	77	1.15 (0.0177)	0.65 (0.0724)	1.53 (0.0241)
15q22	D15S1036	54	1.35 (0.0108)	1.65 (0.0064)	1.65 (0.0186)
**17p12**	**D17S799**	**32**	**2.12 (0.0017)**	**1.65 (0.0064)**	**2.14 (0.0061)**
**18p11**	**D18S1163**	**22**	**1.83 (0.0033)**	**1.05 (0.0282)**	**2.47 (0.0029)**
18p11	D18S453	42	1.18 (0.0164)	0.71 (0.0617)	1.30 (0.0366)
18q12	D18S457	55	1.06 (0.0222)	0.66 (0.0706)	1.17 (0.0487)

MLS was estimated using TWOLOCARP [38], the p value for a single-locus MLS was calculated using "possible triangle restriction" [39], and for a two-locus MLS obtained by Simulation.

NA: not available

Joint IBD sharing probabilities for an affected sibling pair to share 0, 1 or 2 alleles IBD at 6p21 and each additional locus under a general model are shown in Table 2.

**Table 2:** Joint IBD sharing probabilities for an affected sibling pair to share 0, 1 or 2 alleles IBD at 6p21 and each additional locus under a general model.

	IBD sharing at 9p13			IBD sharing at 17p12			IBD sharing at 18p11		
IBD sharing at 6p21	0	1	2	0	1	2	0	1	2

0	0.000028	0.000057	0.057006	0.016443	0.054536	0.038093	0.027815	0.055631	0.027815
1	0.075956	0.151911	0.189911	0.032886	0.180947	0.148061	0.068961	0.137921	0.152572
2	0.117038	0.234076	0.174016	0.063655	0.264517	0.200863	0.057259	0.222837	0.24919

## Discussion

Our study of Finnish affected sib-pairs with type 1 diabetes provides further evidence showing that the genes in the *HLA *region are of the primary importance in this high-risk population. Nevertheless, our results also add to the evidence that there are non-*HLA *loci with suggestive evidence of linkage to three chromosome regions: 9p13, 17p12 and 18p11.

Recently, new methods to carry out whole-genome association study using a large number of single nucleotide polymorphisms (SNPs) have become available. 18p11 has been reported to have robust association in the first genome-wide association study in type 1 diabetes along with several other loci showing a significant association [29]. The evidence of linkage to the chromosome 18p11 in our study was, however, modest, which might provide a support to the findings from the above cited study or be a mere chance. In a large study of 1435 multiplex families the genome-wide linkage analysis of type 1 diabetes found linkage in none of the three non-*HLA *loci as we did [19-26]. Taking into account the small sample size of our current study, we need to be cautious when interpreting our findings.

Current genotyping data originally was used to map loci for nephropathy selecting for statistical analysis only sib-pairs discordant (DSPs) for diabetic nephropathy [30]. For DSPs, linked markers will be characterized by diminished, as opposed to excessive, allele sharing between sibs [31]. Therefore, the lod score peak at 6p21 in affacted sib-pairs with type 1 diabetes, for example, was not revealed in the same region in the DSPs analysis when diabetic nephropathy was considered as the phenotype of interest. This suggests that the findings in the current study were not biased by the presence of nephropathy. However, the findings need to be further examined. The significance of our study is that we reconfirm the major significance of *HLA *genes and suggest that a linkage to chromosome 18p11 region might exist in this population with the highest risk of type 1 diabetes in the world.

## Conclusion

Our genome scan data confirmed the primary contribution of the *HLA *genes also in the high-risk Finnish population, and suggest that non-*HLA *genes also contribute to the familial clustering of type 1 diabetes in Finland.

## Methods

### Patients and families

DNA from 70 Finnish nuclear families with at least two siblings affected with type 1 diabetes, including six families with three and one family with four affected children was collected from Finland. MODY families were not included. A total of 207 individuals (147 sibs and 60 parents) were genotyped, providing with 81 sib-pairs affected with type 1 diabetes for linkage analysis. The original study design was to collect DSPs, siblings affected with type 1 diabetes but discordant for diabetic nephropathy, to map loci for diabetic nephropathy[30]. Therefore, all collected sib-pairs were affected with type 1 diabetes and in each family at least one sibling was affected with nephropathy. All sib-pairs were patients with type 1 diabetes receiving insulin treatment since the onset of the disease. In all affected sib-pairs the first patient was diagnosed before the age of 18 years. Since the patients were ascertained through the national data bases and many of them were diagnosed in the 1970s or the early 1980s, detailed clinical or biochemical data at diagnosis were not available. There is however little doubt that the diabetic patients would have other type of diabetes than type 1. The mean (SD) age at onset of type 1 diabetes in the 147 siblings was 14.5 (10.4) years, ranging from 1.2–53.4 years. Informed consent was obtained from all patients and their parents whose DNA samples were collected. The study was approved by the Ethical Committees of the Finnish National Public Health Institute, Helsinki, Finland and the Karolinska Institute, Stockholm, Sweden.

### Genotyping

DNA was extracted from peripheral lymphocytes, according to standard procedures. A genome wide scan was performed using 900 microsatellite markers and using protocols described by Gretarsdottir *et al.*[32] at the genotyping laboratory of deCODE Genetics, Reykjavik, Iceland. Information of the microsatellite markers and marker positions were obtained from the Marshfield genetic map (Center for Medical Genetics, Marshfield Medical Research Foundation). In the marker set used in this study, the average spacing between markers was ~4 cM, with no gap > 10 cM. Standard PCR techniques with fluorescently labeled primers were used to amplify polymorphic DNA fragments. The PCR products were supplemented with the internal size standard, separated and detected on an Applied Biosystems model 377 Sequencer by use of Genescan version 3.0 peak-calling software. Alleles were called automatically with TrueAllele program (Cybergenetics) and the deCodeGT program was used to fractionate according to quality and to edit the called genotypes [33].

### Linkage analysis

A generalization of maximum lod score (MLS) method proposed by Risch [34] was used to assess the linkage in all affected sib-pairs of each pedigree, which is based on the measurement of the number of alleles (0,1,2) shared identical by descent (IBD) by two affected sibs at a locus. Genehunter (Version 2.0) was first used to estimate a single-locus MLS. We report the nominal p-values for the genome-wide single-locus analysis and note that in order to control family-wise error rate (FWER) one could apply Lander-Kruglyak critical value to obtain adjusted p-value. Nominal p-values in the range of 10^-4 ^were considered a statistically significant threshold for linkage [35]. Threshold MLS-LOD score values at 1.9 and 3.3 were considered for "suggestive" and "significant" linkage, respectively [36].

Two-locus analysis with Risch's method developed via an extension of the method by Cordell [37,38] was then used to estimate the MLS at either a single locus or a second locus conditional on the *HLA *region, where the single-locus MLS was greatest. The null hypothesis for a two-locus analysis is that the locus 2 is not involved in disease, and the results are given for a variety of two-locus models, each fitted with the second locus placed at increments of 1.0 cM across the genome. The multiplicative model estimates the conditional MLS at locus 2, taking account of any effect at locus 1. If loci 1 and 2 are unlinked, the multiplicative conditional MLS for locus 2 will be identical to the single locus MLS for locus 2. The additive and general models calculate the conditional MLS at locus 2 taking account of any effect at locus 1, assuming an additive model for the joint action of loci 1 and 2, or allowing for arbitrary epistasis between loci 1 and 2 in the general model. Significance levels (nominal p values) for a single-locus MLS were calculated according to the possible triangle constraints described by Holmans [39] and for two-locus MLS generated for this particular data set analyzed using simulation [38].

To calculate the two-locus MLS, a prior as well as a posterior probability that each affected sib-pair shares i alleles IBD (i = 0,1,2) at particular positions on the genome is required. For unlinked loci, the IBD probabilities were obtained from the output of Genehunter (version 2.0) [40,41] and for the linked loci on chromosome 6 they were generated allowing for separate male and female recombination fractions using MERLIN [42]. A single-locus MLS for X chromosome was estimated using MAPMAKER/SIBS (version 2.0).

We also estimated the power of our sample to test for linkage using the mean IBD sharing test [43] by computer simulations. Depending on the degree of marker informativity assumed, we estimate that we have 56–72% power to detect at p value 0.01 a locus accounting for a sibling relative risk of 1.8, but only 33–41% to detect a locus accounting for a sibling relative risk of 1.5. The power to detect a locus-specific sibling relative risk of 1.8 at p value 0.001 is also less than 40%. This study clearly has limited power to detect loci of small effect, but it is encouraging that we have found suggestive evidence of linkage at three loci in addition to the significant linkage on 6p21.

## Authors' contributions

QQ participated in the data analysis and drafting of the manuscript; AMÖ and BH took part in the genotyping, managed and analyzed the data and approved the final version of the manuscript; JP and HJC participated in the data analysis and drafting of the manuscript; CS collected the data and approved the final version of the manuscript; LK collected the data and approved the final version of the manuscript; ETW participated in the concept and design of the study and approved the final version of the manuscript; KT and JT were responsible for the conception, funding, design and coordination of the study, approved the final version of the manuscript.
